# Adipokines in Semen: Physiopathology and Effects on Spermatozoas

**DOI:** 10.1155/2018/3906490

**Published:** 2018-06-05

**Authors:** Yaelle Elfassy, Jean-Philippe Bastard, Chloe McAvoy, Soraya Fellahi, Joëlle Dupont, Rachel Levy

**Affiliations:** ^1^Assistance Publique des Hôpitaux de Paris, Hôpital Tenon, Service de Biologie de la Reproduction, Université Pierre et Marie Curie Paris 6, Paris, France; ^2^Sorbonne Universités, UPMC Université Paris 06, INSERM UMRS_938, Centre de Recherche Saint-Antoine, IHU ICAN, Paris, France; ^3^Assistance Publique des Hôpitaux de Paris, Hôpital Tenon, UF Biomarqueurs Inflammatoires et Métaboliques, Service de Biochimie et Hormonologie, Paris, France; ^4^INRA, UMR85, Physiologie de la Reproduction et des Comportements, Nouzilly, France

## Abstract

Adipokines are secreted by adipose tissue and could be the link between obesity and infertility. Different studies investigated the involvement of adipokines in reproductive functions but only a few have looked into the male part. This review assesses adipokine functions on male reproductive parameters. Adiponectin seems to have a positive effect on sperm parameters, whereas other adipokines such as resistin or chemerin would have a rather deleterious effect on spermatogenesis. Semen parameters seem to be impacted when resistin and chemerin are increased: indeed, there is a decrease of sperm motility. Sperm morphology is improved when adiponectin is increased. The most studied adipokine, leptin, has a dual effect with a positive effect on sperm at physiological levels and a negative one for high seminal concentrations. Many semen parameters and fertility itself are disturbed according to semen adipokine levels, even if it is not the only interfering element. Taken together, adipokines are found in human and animal semen and most of them or their receptors are expressed in male genital tract. Although the pathophysiological role of adipokines in semen is not clearly elucidated, the adipokines could influence sperm functionality and could be potential biomarkers of male fertility.

## 1. Introduction

It is well known that adipose tissue is an endocrine organ. It secretes adipokines, which act at endocrine, paracrine, and autocrine levels [1]. These adipokines are not only synthesized and secreted mainly by adipocytes, but also synthesized and secreted by the other cells that make up the adipose tissue, such as macrophages, lymphocytes, and fibroblasts [2, 3]. Moreover, proinflammatory cytokines are secreted mainly by nonadipose cells in adipose tissue [3]. The prevalence of obesity has tripled in the last 30 years [4] in men of childbearing age, which coincides with an increase in infertility that affects currently one in six couples in France (according to the report annual report of the ABM in 2012). Indeed, the Institute of Public Health Surveillance (InVS) found a secular decline in spermatic concentration in the past decades in Western Europe. The link between these two public health problems has been widely described. Studies carried out on large cohorts (1558 men [5] and 526 men [6]) showed a significant correlation between a drop in sperm parameters and an increase in body mass index (BMI) higher than 25 kg/m^2^. The study by Jensen et al. [5] carried out on 1558 men showed a decrease in sperm concentration and count of 21.6% (95% CI: 4.0–39.4%) and 23.9% (95% CI: 4.7–43.2%), respectively, when the BMI was higher than 25 kg/m^2^. In addition, a decrease in sperm motility was observed by an Argentinian team in obese patients (51.4% in the normal BMI group versus 46.6% when BMI was higher than 30, *p* < 0.007) [7]. In 2007, a Chinese study found in the same way a decrease in spermatic parameters (count, concentration, and morphology) in overweight subjects, regardless of circulating concentrations of LH, FSH, estradiol, and testosterone [8]. This suggests that these hormones alone do not explain the association between BMI and sperm parameters. Moreover, obesity is promoted by a positive energy balance, which impacts on the function of the cells involved in spermatogenesis [9]. This hypothesis is reinforced by the results obtained in animal experiments, which showed the existence of a direct relationship between epididymal adipose tissue and fertility, since in rats, the removal of this tissue caused a significant decrease in sperm count [10]. Relationships between circulating concentrations of adipokines and BMI have been widely studied. Indeed, different studies showed a variation of these factors associated with overweightness. Thus, obesity is associated with hyperleptinemia and leptin resistance [11]. In contrast, adiponectinemia decreases in overweight cases [2].

Interestingly, these variations are not definitive since they are reversible after weight loss [12], especially after bariatric surgery. Nevertheless, an association has set up evidence between circulating concentrations of adipokines and sperm quality. Thus, comparing two groups (obese fertile versus infertile men), an Egyptian team observed circulating concentrations of leptin higher in the infertile group compared to the fertile group [13]. It has also been shown that leptinemia was positively correlated with abnormal sperm morphology and negatively correlated with the concentration and sperm motility [13, 14]. This correlation could be the result of the higher circulating leptin levels observed in obese or overweight men leading to a decreased testosterone production by Leydig cells, which is able to interfere with the normal cycle of spermatogenesis [15]. Although it is not an adipokine, ghrelin, a peptide hormone secreted by the stomach which is increased in obesity, is also present in the whole human testis and more particularly in Leydig and Sertoli cells. Its receptors (growth hormone secretagogue receptor (GHS-R)) have been identified in germ cells [15]. In vivo studies demonstrated that ghrelin inhibits the proliferative activity of immature Leydig cells and regulates stem cell factor mRNA expression in rat testis [15]. This hormone in link with fasting is also involved in male fertility. Thus, sperm quality is related to the circulating concentrations of adipokines, but the link with fertility is not currently established.

In addition, the concentrations of adipokines in blood and in seminal plasma are not in the same range. Indeed, adiponectin is 1000 times lower in seminal plasma than in blood, whereas progranulin and visfatin are 100 times more concentrated [2]. The varying concentrations between these two biological fluids suggest a difference in production and a potential action on the surrounding cells (germ cells for sperm). Indeed, several studies carried out in humans and animals showed that most of the adipokines and their receptors are expressed in testis especially in seminiferous tubes and more specifically in Leydig and Sertoli cells and on spermatozoa themselves [16].

Thus, the adipokines of seminal plasma could be privileged actors involved in the relationship between obesity and fertility. Obesity is characterized by an increased number of adipose cells and an excessive storage of triglycerides in the adipose cells. The hormonal interaction between the adipose tissue and other endocrine organs including the gonads is complex and not fully understood. Some endocrine changes involving adipokines could contribute to understand the negative effects of obesity on reproductive function. Many studies have shown the presence and the role of adipokines and their receptors in the female reproductive tract of different species. However, fewer studies have investigated the role of adipokines on male fertility in case of obesity or not, whereas it has consistently been shown that high BMI reduces male fertility. The present review will highlight the location of adipokines in the male genital tract, the molecular mechanisms of action of these molecules, and their potential effect on spermatic parameters in human and animal models when this information is available. Indeed, several adipokines (leptin, adiponectin, resistin, chemerin, visfatin, vaspin, and progranulin) and certain cytokines have already been detected in semen. For adipokines that have been studied thoroughly, we will also report their effects on spermatozoa.

### 1.1. Leptin

#### 1.1.1. Topography in Male Genital Tract and Mechanism of Action

**Figure 1:**
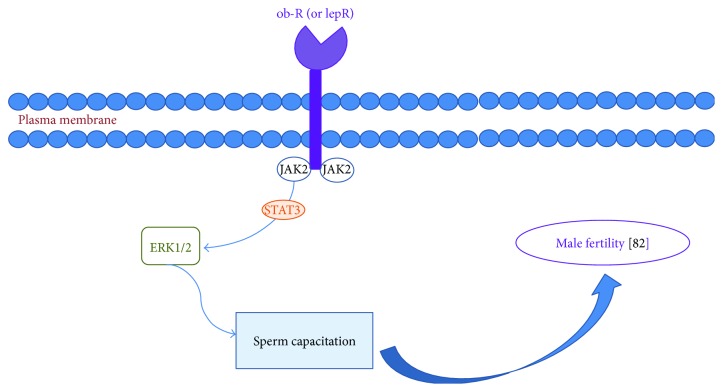
Leptin receptor and its interactions with JAK2 and STAT3 system to sperm capacitation [82].

Leptin is an adipokine mainly secreted by adipose tissue. This hormone of 167 amino acids is encoded by the obese gene (*ob* gene) [17], and its tertiary structure consists of four alpha helices connected by two long and one short loop [18]. This molecule has been widely studied in animals and in humans. Leptin signaling via STAT3 suggests a role in the proliferation of undifferentiated germ cells. Leptin activation of prosurvival pathways may lead to the activation of ERK1/2 signaling, representing capacitation signaling crosstalk (Figure 1). It is intriguing to speculate that acrosomal leptin receptor expression is associated with cholesterol efflux and acrosome reaction, whereas tail leptin receptor expression in human sperm may reflect leptin's modulation of hyperactivated sperm motility. Leptin STAT3 signaling may enable undifferentiated germ cells to replicate without loss of potency while triggering late-stage spermatocytes to undergo development and differentiation [19]. Moreover, leptin modulates the nutritional support of spermatogenesis by human Sertoli cells [15]. Indeed, a Portuguese team demonstrated that acetate production by human Sertoli cells, a central metabolite for spermatogenesis, is severely decreased after exposure to leptin (5 to 50 ng/mL) [9].

Leptin is present in testis and particularly in seminiferous tubules [20]. In animals, studies demonstrate that leptin is expressed differentially between species. Indeed, in pigs, leptin and its receptors are expressed in Leydig cells, whereas in mice, no leptin is present in interstitial cells. In rats, leptin receptor (LepR) mRNA is present in Leydig cells, in Sertoli cells, and possibly in germ cells [21]. Concerning dogs, LepR is absent from Leydig cells and Sertoli cells but present in spermatocytes and spermatids [22]. Aquila [23] has demonstrated the presence of leptin in human sperm at different levels: mRNA expression, protein expression, and immunolocalization. In humans, the presence of leptin receptor has been reported in seminiferous tubules [24]; however, only Jope et al. [25] have reported that seminal plasma and sperm contain this receptor [26]. The presence of leptin receptors on the tail of spermatozoa suggests an effect on motility [25] as described in Section 1.1.2 of this review.

Leptin receptor has also been reported to be present in the sperm of certain species, but there are also reports claiming its absence in other species. Hatami-Baroogh et al., using several commercial and noncommercial antibodies and various techniques, were unable to detect leptin receptors at protein levels in human spermatozoa of fertile (*n* = 22) and infertile (*n* = 50) individuals [27]. Ishikawa reported that in humans, the leptin receptor is present in testicular tissue and confined only to Leydig cells and is not expressed by Sertoli cells, germ cells, or spermatozoa [28]. The difference in the leptin receptor location has been related to species differences.

#### 1.1.2. Effects on Semen Parameters

Although different studies showed contrasting results, it is possible to consider a physiological role of leptin on sperm motility. In fact, studies in which the seminal plasma studied had high concentrations of leptin showed that this adipokine was inversely correlated with sperm motility. Glander et al. [24] showed a negative correlation between seminal leptin and progressive (*r* = −0.53, *p* = 0.0004) and straight (*r* = −0.3, *p* = 0.029) motility for 64 male partners of couples consulting for infertility. This team demonstrated an average leptin concentration in seminal plasma of 2.4 ng/mL, and once the separation into two groups “normozoospermic” and “pathozoospermic”, the mean concentrations of seminal leptin were 1.5 ng/mL and 3.19 ng/mL, respectively. Two other studies have shown a negative correlation between leptin concentrations in seminal plasma and progressive motility. The first study [26] was performed on 79 men with asthenospermia ([leptin] = 4.72 ng/mL) and 77 control men ([leptin] = 3.75 ng/mL). The second study [20] involved 42 infertile patients with varicocele ([leptin] = 3.01 ng/mL) compared to 10 control men ([leptin] = 1.79 ng/mL). It is important to note that a higher concentration of seminal leptin is often associated with spermatic pathologies, suggesting that high concentrations of leptin in seminal plasma would have deleterious effects.

Finally, other studies have concluded that there is no correlation between seminal leptin and sperm motility [29, 30]. In these studies, patients had relatively low leptin concentrations (0.93 ng/mL and 0.95 ng/mL). Despite high concentrations of seminal leptin (5 ng/mL in the nonobese group versus 12.5 ng/mL in the obese group), a South African team found no correlation between seminal leptin and sperm motility [31]. The obese group nevertheless had significantly lower sperm motility than the nonobese group (42.2% versus 54.4% for total motility), and this group had a higher seminal leptin concentration. Thus, analysis of published studies to date suggests that increased seminal leptin concentration would be associated with decreased motility. It can therefore be hypothesized that, at high concentrations, leptin in seminal plasma is associated with a decrease in sperm motility [2]. At lower or “physiological” concentrations, leptin may either have a physiological effect, beneficial to motility, or have no effect.

The same type of result is found when we explore the relationship between seminal leptin and sperm concentration in the ejaculate. Thus, for low concentrations of leptin (0.83–0.91 ng/mL), there is a positive correlation between seminal plasma leptin and sperm concentration (*r* = 0.24, *p* < 0.05) [2]. On the other hand, studies carried out on patients with high seminal concentrations of leptin show a negative correlation between this adipokine and not only the concentration (*r* = −0.187, *p* < 0.05), but also the spermatozoa count (*p* = 0.0001) [20, 32, 33].

Concerning spermatic vitality, two studies did not find any correlation with the seminal levels of leptin [29, 31]. A Chinese team [33], comparing 74 varicocele patients, 70 leukocytospermia patients, and 40 control patients, describes a negative correlation in the case of associated pathology but without supporting their observation by statistical analysis. They showed that patients with varicocele (VC [leptin] = 3.2 ng/mL) and leukocytospermia (LC [leptin] = 2.72 ng/mL) had high concentrations of seminal leptin as well as increased ROS (reactive oxygen species) and apoptosis compared to the control group. They also noted that there was a correlation, for the VC and LC groups, between leptin, apoptosis, and ROS. ROS are markers of oxidative stress, and an increase in these ROS induces a deleterious effect on sperm function [34, 35]. It appears, therefore, that at high concentrations, leptin may be a proapoptotic factor.

The correlation between ejaculate volume and seminal leptin has been poorly studied so far since only two studies presenting contradictory results are at our disposal. On the one hand, Thomas et al. [2] found a negative correlation (*r* = −0.34, *p* < 0.01), whereas Leisegang et al. [31] showed a lack of correlation between levels of seminal leptin and the volume of the ejaculate. It is therefore difficult to decide whether or not there is a link between seminal leptin and ejaculate volume. On the other hand, seminal leptin does not seem to have any effect on sperm morphology since three studies agree on the lack of correlation between these two parameters [2, 29, 31].

To sum up, the analysis of these different studies suggests that there would be an “ideal” seminal concentration of leptin, a concentration at which this adipokine would have a physiological effect, whereas at high concentration, its effects could be deleterious on spermatic parameters. Indeed, at high concentrations, leptin is rather associated with an alteration of certain spermatic parameters, which could have an impact on fertility.

Altered leptin dynamics may contribute to male infertility via at least two mechanisms, both of which may produce hypogonadism. These include leptin resistance or leptin insufficiency at the hypothalamus and leptin modulation of testicular physiology.

#### 1.1.3. Direct Effect on Motility

Leptin may have a physiological role in the male reproductive tract. Thus, an *in vivo* study and an *in vitro* study showed a positive correlation between seminal leptin and motility. The *in vivo* study was performed on 96 men without pathologies associated to spermatogenesis and showed a positive correlation between seminal leptin and progressive (*r* = 0.27, *p* < 0.01) and total (*r* = 0.23, *p* < 0.05) motility [2]. The mean leptin concentrations in seminal plasma were 0.91 ng/mL in normal weight men and 0.83 ng/mL in overweight or obese groups. The *in vitro* work directed by Lampiao and du Plessis [36] aimed to study the effect of leptin on sperm motility: it was shown that after 1, 2, and 3 hours of incubation, leptin significantly increased the total and progressive motility (*p* < 0.05). This study was performed on spermatozoa from normozoospermic donors.

On buffalos, Khaki's team conducted two types of protocols. In the first one, they added 10 ng/mL of leptin on spermatozoa in semen, which was shown to preserve motility and vitality during frozen sequence compared to the control group. For the second protocol, they added 200 ng/mL which had a deleterious effect on semen parameters [37]. This deleterious effect at high levels of leptin consolidates the dual effect of leptin according to the concentration. In male mice, diet-induced obesity induces not only significant impairments of sperm function parameters, but also disruption of the blood-testis barrier integrity [38]. Even if it has been shown that leptin can cross the blood-testis barrier [39], we can hypothesize that obesity could also facilitate the passage of leptin and other adipokines through this barrier.

#### 1.1.4. Transgenic Animal Model (cf Table 1)

A recent study showed testicular atrophy in an *ob/ob* mice model, with testis weight 13% less than the control group (*p* < 0.0001) despite higher body weight [40]. Likewise, this model displayed a decrease in the nuclear volume of Sertoli cells, spermatogonia, and spermatocytes. The same transgenic mice model already demonstrated these effects in 2006 [41]. Furthermore, leptin treatment of adult *ob/ob* males corrects their sterility, an effect that is mediated at least partly by a normalization in testicular weight, spermatogenesis, and Leydig cells morphology [42].

It was shown that LepR gene null mice generate infertile phenotype [43] and present a decrease of gonadal functions [44].

Male *ob/ob* mice are morbidly obese and infertile. Similar phenotypes are observed in Lep-R-deficient mice and the Zucker fatty (fa/fa) rat. Pubertal obese Zucker rats present altered spermatogenesis, as observed in the histological level, which persists up to the adult phase; in the quantitative analysis, sperm production in the fatty animals was reduced as well, but only in the pubertal rats. On the other hand, the increased sperm DNA fragmentation found in the adult rats points out genetic damage generated in the fatty rat gamete, which can be a lead for understanding the obese Zucker rat's infertility [45].

#### 1.1.5. Polymorphisms in Human

Different polymorphisms of leptin do exist and are characterized in many studies. Leptin rate is more increased in AA genotype than in AG genotype [46]. LEP-2548G/A genotype is different between fertile and infertile patients (*p* = 0.012). AA genotype is increased in the infertile group, and AG genotype decreased in this group, which induces that AG genotype has a protective/safety effect on fertility by reducing the risk of male infertility by 3-fold [47]. Sperm count is increased in the infertile group with AG and GG genotypes than AA (*p* = 0.0009 and *p* = 0.026). Leptin's receptor polymorphisms exist too and influence spermatic motility so that progressive motility is increased in RR genotype than QQ and QR ones [47].

All this data supports a local role for leptin in sperm parameters with the consequent potential impact on fertility capacity.

### 1.2. Adiponectin

#### 1.2.1. Topography in Male Genital Tract and Mechanism of Action

Adiponectin is a protein of 224 amino acids mainly produced by white adipose tissue but also found in other tissues such as bone and muscle [16]. Unlike the majority of adipokines, plasma adiponectin concentration is negatively correlated with BMI and visceral adiposity [48], but it also regulates gonadotropic axis and gonad function [49]. Adiponectin is found in the circulation in various molecular forms: the so-called LMW (low molecular weight) form corresponding to an assembly of 3 adiponectin monomers in trimers, the MMW (medium molecular weight) form corresponding to hexamers (assembly of 2 trimers), and the form called HMW (high molecular weight) which corresponds to an assembly of 3 hexamers [50]. The HMW form of adiponectin is the predominant circulating form (>80%) and would be the most active. Most studies published to date have been conducted with total adiponectin, but some have been performed with the dosage form HMW.

Adiponectin mRNA is present in testis and has been found in Leydig cells [51] and spermatocytes [52]. AdipoR1 and AdipoR2 (adiponectin receptor) are present in testis [53, 54] more particularly in seminiferous tubules and specifically in interstitial tissue of rats [48]. Indeed, these receptors are abundant in Sertoli cells, Leydig cells, and germ cells in rats. Kawwass et al. also reported a presence of adiponectin receptors on spermatozoa themselves [54]. Adiponectin and adiponectin receptors have been immunolocalized on bull's spermatozoa in acrosomal, postacrosomal, equatorial, and tail regions [55].

Adiponectin protein is abundant in the tail region of bull sperm, while AdipoR1 is localized mainly at the equatorial and acrosome region and AdipoR2 is expressed primarily on the sperm head region and on the equatorial line. Adiponectin and its receptors are expressed during pre- and postcapacitation of spermatozoa, suggesting that adiponectin might have a role in sperm capacitation [55]. Thus, local actions of adiponectin in testis are involved in the production of sperm capable of fertilization [56].

#### 1.2.2. Effects on Semen Parameters

To our knowledge, only one team until now [2] has studied the relationship between seminal adiponectin concentrations (total adiponectin form) and sperm parameters in humans. This study suggested that adiponectin would rather have a positive effect on sperm function. Mean seminal adiponectin concentrations of 16.8 ng/mL and 14.2 ng/mL (a thousandfold lower than adiponectinemia) were measured in normal weight subjects and in overweight or obese patients, respectively.

Adiponectin levels in seminal plasma have been shown to be positively correlated with sperm concentration, sperm count, and percentage of typical sperm forms.

**Figure 2:**
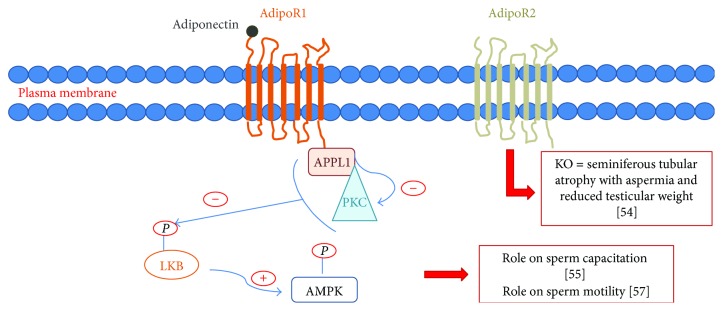
Adiponectin receptors and its possible interactions to fertility.

An animal study showed an improvement of fertility in bulls positively correlated with the seminal concentration of adiponectin (*r* = 0.80, *p* < 0.0001) and its AdipoR1 receptors on spermatozoa (*r* = 0.90; *p* < 0.0001) and AdipoR2 (*r* = 0.65, *p* < 0.0001) [51]. After capacitation, the levels of adiponectin and its receptors are lowered, suggesting a direct role on sperm motility. Interestingly, a novel association of adiponectin system with sperm motility was shown in rams [57] (Figure 2).

#### 1.2.3. Transgenic Animal Model (Table 1)

An adiponectin receptor gene knockdown study performed in mice highlighted the potential importance of the adiponectin pathway in the male genital tract. Indeed, this work has shown that the loss of AdipoR2 was responsible for seminiferous tubular atrophy associated with aspermia and reduced testicular weight [54] (Figure 2). Moreover, a decrease of testis weight was evidenced by Bjursell et al. with the same model [58].

It seems that adiponectin may play a beneficial role in male reproductive function, but this pathway has yet to be studied and confirmed. There are no *in vitro* studies carried out on seminal adiponectin.

#### 1.2.4. Polymorphisms in Human

Polymorphism has only been described for females, to the best of our knowledge with a link to insulin resistance in polycystic ovary syndrome patients [59]. However, no polymorphism has been described with a link to male infertility.

### 1.3. Resistin

#### 1.3.1. Topography in Male Genital Tract and Mechanism of Action

Resistin is a 12.5 kDa adipokine belonging to a family of cysteine-rich proteins [16]. It is present in testis, in seminiferous tubules, and specifically in Leydig and Sertoli cells [60] of animals, but this has not been demonstrated in humans. TLR-4, a binding site for resistin, has been found in human sperm [16].

#### 1.3.2. Effects on Semen Parameters

To our knowledge, only three studies have measured resistin in seminal plasma. The team of Moretti et al. showed that there was a negative correlation between the concentrations of seminal resistin and spermatic motility and vitality [61]. Two other teams [2, 62] studied the relationships between resistin concentrations in seminal plasma and sperm parameters but did not show significant correlation. However, given the low number of studies available, it is difficult to conclude on the role of resistin, which seems to have a rather negative effect on spermatozoa and thus fertility.

However, it has been shown that this adipokine is associated with markers of inflammation in seminal plasma. Indeed, the concentrations of seminal resistin correlate positively with those of proinflammatory mediators such as elastase, interleukin-6 (IL-6) [62], and tumor necrosis factor-*α* (TNF-*α*) [61]. During inflammation, the concentrations of cytokines and ROS increase, and this may have a deleterious effect on the male reproductive function [63, 64]. Indeed, it has been shown that an increase in ROS could induce a decrease in spermatic concentration, motility, and sperm count [34]. In the study published by Moretti et al., the seminal concentrations of resistin were significantly higher in cases of leukocytospermia or if the patients were smokers [61]. This increase in resistin concentrations was also associated with a significant increase in TNF-*α* and IL-6, as well as a sharp decrease in spermatic motility and the number of normal morphology spermatozoa for patients with leukocytospermia. All these results suggest that resistin could be considered as a marker of inflammation, and in pathological situations such as leukocytospermia, the presence of this adipokine would be related to an alteration of sperm parameters.

#### 1.3.3. Polymorphisms in Human

As for adiponectin, we found that resistin polymorphism has only been described for females and more particularly in cases of polycystic ovary syndrome [65].

### 1.4. Chemerin

#### 1.4.1. Topography in Male Genital Tract and Mechanism of Action

Chemerin, a recently discovered adipokine, is synthesized mainly by the liver, kidney, and adipose tissue [66]. Few studies have been carried out on this adipokine and in particular on its role in the reproductive function. In human as in rodents, chemerin receptors (CMKLR1, GRP1, and CCRL2) are present in testis. Chemerin, CMKLR1, and GPR1 are localized specifically on Leydig cells and poorly on germ cells [16].

#### 1.4.2. Effects on Semen Parameters

To our knowledge, only one study was carried out in humans for this adipokine [2]. Chemerin was detected in the seminal plasma of 96 men with no spermatogenesis abnormalities, and it was shown that this adipokine correlated negatively with spermatic motility and positively with sperm concentration. Thomas' team [2] showed increased chemerin concentrations in the sperm of control subjects compared to a group of vasectomized patients (*p* < 0.001). This data suggests that there would be a local secretion of chemerin in the male genital tract, particularly at the testicular level.

#### 1.4.3. In Vitro Experiment

**Figure 3:**
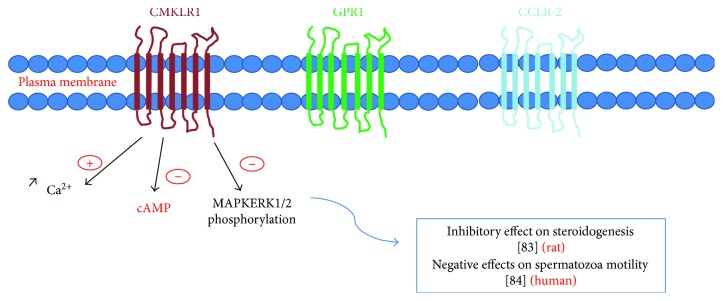
The three transmembrane receptors (CMKLR1, GPR1, and CCLR-2) and its known interactions to male fertility [83, 84].

Surprisingly, it was demonstrated by experiments conducted *in vitro* on rats that chemerin had an inhibitory effect on steroidogenesis [16] (Figure 3). The roles played by this adipokine in human semen needs to be further investigated.

### 1.5. Visfatin

Visfatin, also known as NAMPT, is a recently discovered adipokine produced primarily by perivascular adipose tissue. It has been found in Leydig cells, spermatocytes, and spermatozoa [16]. Visfatin levels are a hundred times higher in seminal plasma than in blood suggesting a significant local production in the male genital tract [2, 16].

No other studies are available to further understand the effects of this adipokine on male fertility.

### 1.6. Vaspin

Vaspin, another recently discovered adipokine, is expressed in epididymal, retroperitoneal, and mesenteric adipose tissue and is related to the metabolic state [67]. Thomas et al. showed that seminal plasma vaspin was negatively correlated with ejaculate volume (*r* = −0.36, *p* < 0.001) and positively correlated with sperm DNA fragmentation (*r* = 0.22, *p* < 0.05) [2].

### 1.7. Progranulin

Progranulin is increased in cases of obesity or metabolic syndrome and could contribute to the inflammatory mechanisms found in certain pathologies via a recruitment of macrophages [68]. This adipokine was studied in the seminal plasma only by Thomas et al. [2]. Progranulin is positively correlated with motility (*r* = 0.32, *p* < 0.001), sperm count (*r* = 0.23, *p* < 0.05), and sperm morphology (*r* = 0.25, *p* < 0.01). In vasectomized patients, seminal progranulin levels were significantly decreased (*p* < 0.05), indicating probable local secretion.

### 1.8. Cytokines

#### 1.8.1. Topography in Male Genital Tract and Mechanism of Action

In the wide family of cytokines, some have been described in semen and related to male fertility. The presence of tumor necrosis factor- (TNF-) *α* and interferon- (IFN-) *γ* will be further investigated here. In dogs, TNF is present in testis (more particularly in germ cells, but not in Sertoli cells nor Leydig cells), epididymis, and spermatozoa [69]. Proinflammatory cytokine like TNF-*α* can directly impair the seminiferous epithelium by damaging the expression and assembly of the junctional proteins leading to an impairment of the blood-testis barrier [70]. Moreover, proinflammatory cytokines disrupt the seminiferous and epididymal epitheliums by creating high levels of ROS [70].

#### 1.8.2. Effects on Semen Parameters

Different authors report that cytokine levels are increased in the seminal plasma of infertile male [71–73]. It is the case for TNF-*α* and IFN-*γ* that rise in semen from males with an inflammation linked to infertility [74].

On the one hand, cytokines seem to have a bad effect on sperm motility [75, 76]. This was confirmed by Paradisi that showed a negative correlation between TFN-*γ* and sperm concentration, motility, and morphology [73]. In 2013, it was also confirmed that TNF-*α* levels are increased in the seminal plasma of oligozoospermic (42%, *p* < 0.01) and asthenospermic patients (58%, *p* < 0.001) compared to control patients. On the other hand, one study did not find any effect of either TNF-*α* or IFN-*γ* on sperm motility [77].

#### 1.8.3. In Vitro Experiments

One study observed *in vitro* effects of TNF and IFN on spermatozoa for 3 hours and showed a decrease of 18% of sperm motility between 60 and 180 minutes and a decrease of 16% of sperm vitality at 180 minutes [78].

#### 1.8.4. Polymorphisms in Human

A polymorphism in the TNF-*α*_308 gene was associated with a significant decrease of sperm count, sperm motility, normal sperm morphology, and acrosin activity [79]. In the same study, the occurrence of A allele was significantly increased in infertile patients than fertile controls (21.6% versus 9.7%; OR: 0.388, *p* = 0.005). An AA genotype of TNF-*α* corresponds more to a lowering concentration, motility, and normal morphology sperm profile. Moreover, TNFR-1 36G allele is more found in oligozoospermia associated to a decrease of sperm concentration [80].

## 2. Discussion and Conclusion

Taken together, leptin is the most studied adipokine in male fertility; fewer data are available for the other adipokines. For example, until now, it is unclear if adiponectin, resistin, visfatin, vaspin, progranulin, and chemerin are able to cross the blood-testis barrier. Leptin is present in germ cells, but there is no consensus for the presence of its receptor on sperm. It could depend on spermatozoa origin because Jope et al. found it on ejaculated spermatozoa [25], whereas Ishikawa researched it on spermatozoa obtained directly from the testis and could not found any LepR on these sperms [28]. Otherwise, the other article, which concluded to a lack of LepR on spermatozoa, found nonetheless by RT-PCR LepR on 1 of 10 controls and 3 of 23 infertile patients [27]. Thus, these two articles have to be discussed cautiously and checked on ejaculated sperm. Our point of view is that LepR would appear on mature spermatozoa. We promote the dual role of leptin according to its concentration in seminal plasma. We hypothesize a beneficial role of leptin at physiological levels as we can expect it in men with normal BMI. On the contrary, a negative effect of leptin on spermatozoa is suggested for high concentration, corresponding to those determined in overweight or obese men. The mechanism of action of leptin on spermatozoa could be direct because human receptor of leptin has been found on spermatozoa itself. However, it also could be the consequence of higher circulating levels of leptin in obese or overweight men leading to a decrease of testosterone production by Leydig cells, which therefore interferes with the normal cycle of spermatogenesis [15]. Moreover, leptin can also modulate the nutritional support of spermatogenesis by human Sertoli cells [15]. Indeed, exposure of human Sertoli cells to leptin dramatically decreases the production of acetate, which is a central metabolite for spermatogenesis [9].

In animals and humans, adiponectin is less concentrated in seminal plasma than in serum by 180-fold in bulls [81] and 66-fold in humans [2]. Different isoforms of adiponectin circulate in blood, with a large predominance of HMW adiponectin. One hypothesis of this huge difference of concentration between these two fluids is a possible crossing of the blood-testis barrier only by smaller molecules. Even if Heinz's team got proportionally more HMW adiponectin in semen than other forms, which reduce this hypothesis, it is also reported that Ca^2+^ is 3-fold more concentrated in semen than blood and can promote HMW adiponectin forming from small isoforms that come from blood leakage. Adiponectin's effects on spermatozoa seem to be beneficial, which is in agreement with a better fertility in lean men.

Concerning the other adipokines described in the present review, only one study reported their concentration in human seminal plasma in normal weight, overweight, and obese patients. Even if these data need to be confirmed, it is clear that adipokines might be a link between obesity and male infertility. It would be worthwhile to determine seminal adipokines and adipokines expression in testis cells in some pathologies of male genital tract.

The increase of resistin and doubtlessly many other adipocytokines involved in inflammation in seminal plasma is correlated with a decrease of sperm vitality and motility. We need more in vitro experiments to assess the ideal physiologic concentrations of each adipokine and their synergic effects on spermatogenesis and sperm fertilization capacity. We cannot find enough information on combinatory actions of adipokines on male fertility and semen parameters, whereas all these adipokines are present in seminal plasma and many should be increased concomitantly and could interact together. Also, since some circulating adipokines like adiponectin can be modulated by nutrition, it will be very interesting to investigate if dietary supplements could affect seminal adipokines or adipokine testis expression and consequently improve male fertility. More experiments to assess the best levels of each adipokine and synergic effects of these hormones on spermatogenesis and sperm fertilization capacity are necessary.

In conclusion, some adipokines have been found in human and animal semen. Studies performed *in vitro* and *in vivo* by using transgenic animal models confirmed the adipokine's effects observed on semen parameters. Thus, adipokine profiles in seminal plasma could be a biomarker of male fertility. It could be interesting to measure these markers in the semen of infertile men to evaluate their seminal metabolic profile.

## Figures and Tables

**Table 1:** Consequences on male fertility phenotypes of animal models with missing adipokine or adipokine receptor.

Type	Phenotype	References
*ob/ob* mice	Testicular atrophy Decrease nuclear volume of Sertoli cells, spermatogonia, and spermatocytes	[40]
*ob/ob* mice	Infertile Reduction of testis weight, multinucleated spermatids, few spermatozoa, and anormal Leydig cells	[41]
*db/db* mice	Infertile	[43]
*db/db* mice	Infertile Impairment of spermatogenesis and sperm motility	[14]
*fa/fa* rat (Zucker rat)	Alteration in sperm production and sperm DNA damage	[45]
AdipoR2	Seminiferous tubular atrophy with aspermia and reduced testicular weight	[54]
AdipoR2	Decrease of testis weight	[58]
